# The Matrine Derivative MASM Alleviates LPS-Induced Depressive-Like Behavior in Mice by Modulating Hippocampal Inflammation, Oxidative Stress, and Autophagy

**DOI:** 10.31083/AP47820

**Published:** 2025-10-30

**Authors:** Chao-Ran Li, Zhang-Yang Xu, Lu-Na Sun, Ling-Ling Wu, Wen-Feng Zeng, Jie Zhou, Yong-Jie Lian, Yan Wang, Yun-Xia Wang

**Affiliations:** ^1^Department of Nautical Psychology, Faculty of Psychology, Naval Medical University, 200433 Shanghai, China; ^2^School of Pharmacy, Naval Medical University, 200433 Shanghai, China

**Keywords:** MASM, BV2 cell, microglia, neuroinflammation, oxidative stress, autophagy

## Abstract

**Background:**

Previous studies have demonstrated a significant association between neuroinflammation and major depressive disorder (MDD). (6aS,10S,11aR,11bR,11cS)-10-methylamino-dodecahydro-3a,7a-diaza-benzo(de)anthracene-8-thione (MASM), a derivative of matrine, has recently been shown to display anti-inflammatory properties. However, its effects on lipopolysaccharide (LPS)-induced depression and the underlying mechanisms remain unexplored. This study aimed to assess the effects of MASM on depressive-like behaviors induced by LPS and to investigate the potential mechanisms involved.

**Methods:**

Following intraperitoneal injection of LPS (0.83 mg/kg), MASM was administered. Depressive-like behaviors were assessed through the forced swim test (FST) and tail suspension test (TST). To further explore the mechanisms, LPS-induced BV2 microglial cell models were established. Enzyme-linked immunosorbent assay (ELISA) was used to quantify the expression of TNF-α and high mobility group box 1 (HMGB1), while immunoblotting was performed to assess heme oxygenase-1 (HO-1), sirtuin 1 (SIRT-1), p62, and microtubule-associated protein 1A/1B-light chain 3-phosphatidylethanolamine conjugate (LC3-II) expression. Reactive oxygen species (ROS) levels were evaluated using flow cytometry.

**Results:**

MASM pretreatment markedly ameliorated acute depressive-like behaviors in LPS-treated mice and upregulated HO-1 expression in the hippocampus. In LPS-stimulated BV2 cells, MASM reduced the levels of proinflammatory markers TNF-α and HMGB1. Furthermore, MASM mitigated LPS-induced oxidative stress, as evidenced by increased ATP, HO-1, and SIRT-1 levels, along with decreased ROS levels. MASM also restored autophagic function, demonstrated by increased LC3-II expression and reduced p62 levels.

**Conclusion:**

These findings suggests that MASM alleviates LPS-induced neuroinflammation and acute depressive-like behaviors, possibly by reducing oxidative stress and promoting autophagy.

## Main Points

∙ Administration of the matrine derivative (6aS, 10S, 11aR, 11bR, 11cS)-10-methylamino-dodecahydro-3a, 7a-diazabenzo [de] anthracene-8-thione (MASM) ameliorated lipopolysaccharide (LPS)-induced depressive-like behaviors.

∙ MASM treatment attenuated neuroinflammation via the reduction of TNF-α and high mobility group box 1 (HMGB1) in BV2 cells.

∙ MASM treatment altered heme oxygenase-1 (HO-1), sirtuin 1 (SIRT-1), ATP, and reactive oxygen species (ROS) expression under stress conditions.

∙ MASM treatment enhanced autophagy in BV2 cells.

## 1. Introduction

Major depressive disorder (MDD) is a common mood disorder, primarily characterized by persistent low mood, a marked reduction in interest or pleasure in activities, and, in severe cases, may result in suicide [1]. MDD affects millions globally, imposing a substantial burden on both families and society, as well as negatively impacting public health and economic development. Although there have been significant advances in both pharmacological and psychological treatments, the response rate remains limited, with efficacy observed in only 60%–70% of patients [2]. Additionally, the delayed onset of therapeutic effects and adverse side effects, such as nausea, insomnia, and sexual dysfunction [3], limit the clinical utility of conventional antidepressant drugs. Therefore, there is an urgent need to develop alternative therapies that are both effective and safe.

Substantial evidence has established a link between depression and chronic inflammation [4]. Overproduction of proinflammatory cytokines is crucial to the initiation and progression of depression [5, 6]. Elevated levels of tumor necrosis factor-alpha (TNF-α), interleukin-6 (IL-6), and interleukin-1 beta (IL-1β) have been frequently observed in the bloodstream of MDD patients [7]. In addition to peripheral inflammation, studies have demonstrated that inflammation within central brain regions, such as the hippocampus, also plays a role in the development of depression [8, 9]. Microglia, the immune cells residing in the central nervous system (CNS), become activated in response to tissue damage and protect against pathogens [10]. Under stressors such as psychological or physical stimuli, the activation of microglia results in the release of inflammatory cytokines, leading to sustained low-grade inflammation in the hippocampus and medial prefrontal cortex [11, 12]. This disruption further affects the kynurenine pathway while promoting excessive accumulation of reactive oxygen species (ROS) and suppressing autophagy resulting in MDD [13].

Matrine, an active alkaloid compound extracted from *Sophora flavescens* [14], has been employed in traditional Chinese medicine for its various pharmacological effects, including immunomodulatory, anti-inflammatory, and anti-fibrotic properties [15, 16, 17]. Nonetheless, its clinical application is limited by its low therapeutic effectiveness. To address this limitation, various matrine derivatives have been developed, among which MASM [(6aS,10S,11aR,11bR,11cS)-10-methylamino-dodecahydro-3a,7a-diazabenzo [de] anthracene-8-thione] has demonstrated enhanced anti-inflammatory effects *in vitro*. Previous studies have underscored the anti-neuroinflammatory potential of MASM [18]. For example, MASM treatment has been shown to inhibit astrocyte reactivity and preserve astrocytic function in experimental autoimmune encephalomyelitis [19]. Furthermore, Xu *et al*. [20] reported that MASM suppresses LPS-induced inflammation and functional maturation of murine bone marrow-derived dendritic cells. However, the anti-inflammatory effects of MASM within the context of depression and microglial activation have not been investigated. Therefore, this study aimed to evaluate the antidepressant effects of MASM and elucidate the underlying mechanisms.

Initially, we established a lipopolysaccharide (LPS)-induced mouse model to assess the effects of MASM on alleviating acute depressive-like behaviors and heme oxygenase-1 (HO-1) expression *in vivo*. Subsequently, we investigated the impact of MASM on oxidative stress and autophagy *in vitro* using LPS-treated microglial BV2 cells. Our findings suggest that MASM may attenuate the LPS-induced neuroinflammatory response and acute depressive-like behaviors by improving oxidative status and enhancing autophagic activity in microglia.

## 2. Materials and Methods

### 2.1 Reagents

MASM (purity >99%) was synthesized by the Department of Organic Chemistry, School of Pharmacy, Naval Medical University. Lipopolysaccharide (LPS; derived from *Escherichia coli* O127, Cat# L3129) was sourced from Sigma Aldrich (Saint Louis, MO, USA). High-glucose Dulbecco’s Modified Eagle Medium (DMEM; Cat# 10569010), fetal bovine serum (FBS; Cat# 10099-141), penicillin-streptomycin (Cat# 15140-122), and 0.25% trypsin-EDTA (Cat# 25200-072) were obtained from Gibco (Thermo Fisher Scientific, New York, NY, USA). Antibodies used in this study included anti-HO-1 (Cat# 10701-1-AP) and anti-p62 (Cat# 18420-1-AP) from Proteintech (Wuhan, Hubei, China), anti-β-actin (Cat# AC026) from ABclonal (Wuhan, Hubei, China), anti-β-tubulin (Cat# 5568S) and anti-sirtuin 1 (SIRT)-1 (Cat# 9475S) from Cell Signaling Technology (New York, NY, USA), and anti-microtubule-associated protein 1 light chain 3 (LC3) (Cat# 381544) from Zenbio (Chengdu, Sichuan, China).

### 2.2 Animals and Experimental Design

Thirty male BALB/c mice, aged 7 weeks, were obtained from the Experimental Animal Center at Naval Medical University (Shanghai, China). The mice were housed in groups under standard laboratory conditions, maintained at a constant temperature of 22 °C, with 52% relative humidity, and a 12-hour light/dark cycle. Food and water were provided ad libitum. All experimental procedures were reviewed and approved in accordance with the guidelines issued by Naval Medical University.

After a 7-day acclimation period, the mice were randomly assigned to three groups: control group, LPS group, and LPS+MASM group. To induce an acute depressive-like behavior model, LPS (0.83 mg/kg) [21] was administered intraperitoneally at 19:00 on the experimental day. MASM (0.25 mg/kg) was given intraperitoneally 2 hours before the LPS injection. The control group received an intraperitoneal injection of saline. Behavioral assessments, including the tail suspension test (TST), forced swim test (FST), and open field test (OFT), were conducted at 19:00 on the day following LPS administration. After the behavioral tests, the mice were euthanized using tribromoethanol (0.2 mL/10 g), and their hippocampi were rapidly dissected and stored at –80 °C for subsequent analyses.

### 2.3 Behavioral Tests

After 1 day of drug administration, all mice were given a 24-hour rest period. Subsequently, following weighing, behavioral tests were conducted during the dark phase between 19:00 and 22:00.

#### 2.3.1 Tail Suspension Test (TST)

The Tail Suspension Test is a well-established method for assessing behavioral manifestations of despair and helplessness in murine models [21]. The test was performed using an automated TST device (MED-TSS-MS, MED Associates Inc., St. Albans, VT, USA). Prior to each trial, the inner walls of the chambers were cleaned with 75% ethanol (C069156931, Nanjing Regent, Nanjing, Jiangsu, China). The test duration was 6 minutes, consisting of a 1-minute adaptation period followed by a 5-minute testing period. Data were analyzed using Tail Suspension SOF-821 software (MED Associates Inc., St.). Consistent with previous studies, an immobility threshold was set at 0.75, with signals below this threshold considered indicative of immobility. The cumulative duration of immobility was recorded as the primary measure.

#### 2.3.2 Forced Swimming Test (FST)

The Forced Swimming Test is widely used to evaluate depressive-like behavior in animals [22]. The FST detection system (SuperFst high-throughput FST system, Xinruan, Shanghai, China) analyzed the activity of mice through video recording and grayscale tracking. This system distinguishes between floating, swimming, and struggling behaviors and calculates the total floating time, which is considered as immobility time. Mice were gently placed in a water container filled with water at 25 °C to a depth of approximately 16–18 cm. The total recording time was 6 minutes, and the immobility time during the last 5 minutes was used for analysis.

#### 2.3.3 Open Field Test (OFT)

The Open Field Test assesses autonomous activity and anxiety in animals [23]. The test was conducted in a square, opaque box measuring 42 × 42 × 42 cm (RD1112-IOF, Shanghai Transfer Info Technology, Shanghai, China). A camera positioned at the top of the box recorded the mice’s movement, and activity trajectories were analyzed through video recording and grayscale tracking. The total distance traveled and the distance traveled in the central zone were calculated. The experiment lasted 6 minutes, with the data from the last 5 minutes used for analysis.

### 2.4 Cell Culture and Treatment

The BV2 microglial cells were obtained from the China Center for Type Culture Collection (CCTCC, Wuhan, Hubei, China), a certified and reputable cell bank. All cell lines were validated by short tandem repeat (STR) profiling and confirmed negative for mycoplasma. The cells were cultured in high-glucose DMEM supplemented with 10% fetal bovine serum (FBS) and 1% penicillin-streptomycin and maintained at 37 °C in a 5% CO_2_ incubator (Huachen High Pressuer Vesssel Group Cooperation, Jinan, Shandong, China). Subculturing was conducted at a 1:3 ratio, with only passages up to the sixth used for experiments. The cells were assigned to six groups: control, LPS (model), LPS+MASM (10 µM), LPS+MASM (20 µM), MASM (10 µM), and MASM (20 µM). For the ROS assays, an additional group (LPS+MASM 50 µM) was included. This group was added to evaluate the potential dose-dependent effects of MASM on ROS levels. Cells in all groups were stimulated for 6 hours based on preliminary findings indicating a significant increase in intracellular ROS levels at this time point. BV2 cells were seeded at a density of 1 × 10^5^ cells/mL and allowed to adhere for 24 hours. Following this, the cells were pretreated with MASM (10 or 20 µM) for 2 hours before exposure to LPS (1 µg/mL). After an additional 24-hour incubation, both the cells and supernatants were collected for further analysis.

### 2.5 ROS Assay

The intracellular concentration of reactive oxygen species (ROS) was quantified using a commercially available DCFH-DA kit (Cat# S0033S, Beyotime, Shanghai, China). Cells were incubated with the DCFH-DA working solution for 30 minutes. Following incubation, the culture medium was aspirated and cells were resuspended in PBS. Subsequently, flow cytometry (CytoFLEX, Beckman Coulter, Pasadena, CA, USA) was employed to measure fluorescence intensity at an excitation wavelength of 488 nm and an emission wavelength of 525 nm, which served as an indicator of ROS levels.

### 2.6 Enzyme-Linked Immunosorbent Assay (ELISA)

The levels of TNF-α (Cat#F11630) and high mobility group box 1 (HMGB1) (Cat#F10620) were determined using commercial kits provided by Westang (Shanghai, China), following the protocols outlined by the manufacturer. The absorbance values were then computed based on the standard curves.

### 2.7 Western Blotting

The hippocampal tissue and BV2 cells were lysed in a lysis buffer and homogenized. Protein concentrations were measured using a bicinchoninic acid (BCA) protein assay kit (P0010, Beyotime). Equal protein amounts were loaded onto an SDS-PAGE gel (PG112, epizyem techonology, shanghai, china) for separation and then transferred to polyvinylidene fluoride (PVDF) membranes (FFP39, Beyotime) through electroblotting. After blocking with bovine serum albumin (BSA), the PVDF membranes were incubated overnight at 4 °C with primary antibodies, including anti-HO-1 (1:1000), anti-p62 (1:1000), anti-β-tubulin (1:1000), anti-SIRT-1 (1:1000), anti-LC3 (1:1000), and anti-β-actin (1:100,000). Subsequently, membranes were incubated with an IRDye-conjugated secondary antibody (1:10,000, Cat# 926-32211, Li-COR Biosciences, Lincoln, NE, USA) for 1 hour at room temperature. Protein bands were detected using the Odyssey imaging system (Odyssey 3198, LI-COR, Lincoln, NE, USA) and analyzed using ImageJ 1.53e software (National Institutes of Health, Bethesda, MD, USA). The expression levels of the proteins were normalized to either β-tubulin or β-actin.

### 2.8 Statistical Analysis

Data analysis was conducted using SPSS software version 26.0 (IBM, Armonk, NY, USA) and data visualization was performed with GraphPad Prism version 8 (GraphPad Software, Inc., Boston, MA, USA). Statistical comparisons were made using a one-way analysis of variance (ANOVA) followed by the least significant difference (LSD) post-hoc test. A *p*-value of less than 0.05 was considered statistically significant. The results are expressed as the mean ± standard error of the mean (SEM).

## 3. Results

### 3.1 MASM Attenuated LPS-Induced Depressive-Like Behaviors

**Fig. 1.**
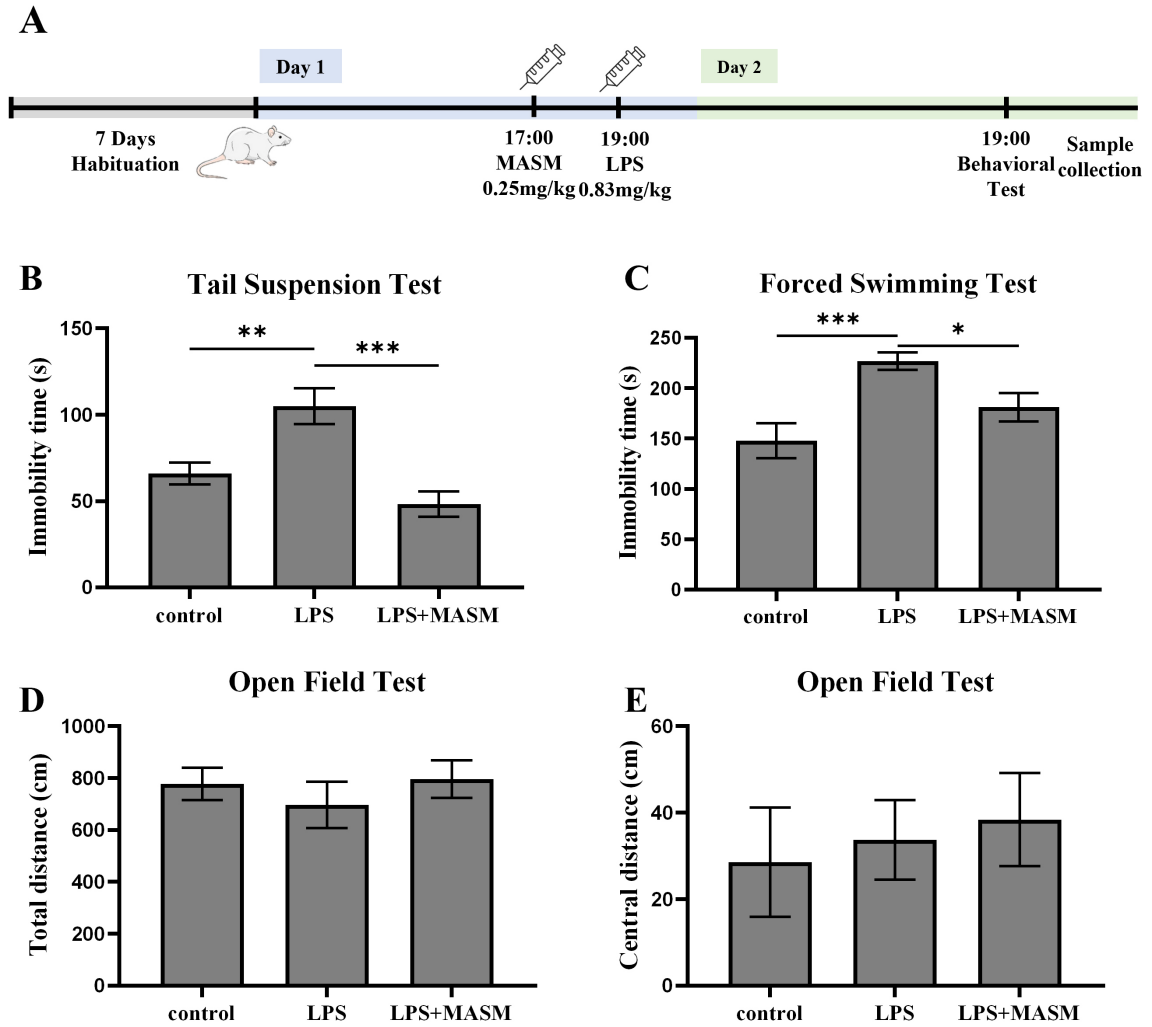
**MASM mitigates LPS-induced depressive-like behaviors**. (A) Experimental procedure timeline. (B) Tail Suspension Test (TST) (one-way ANOVA followed by LSD’s multiple comparison tests, F _(2,24)_ = 12.593, n = 9, *p* < 0.001; Control vs LPS, *p* = 0.002; LPS vs LPS+MASM, *p* < 0.001). (C) Forced Swimming Test (FST) (one-way ANOVA followed by LSD’s multiple comparison tests, F _(2,21)_ = 8.205, n = 8, *p* = 0.002; Control vs LPS, *p* < 0.001; LPS vs LPS+MASM (0.25), *p* = 0.030). (D,E) Open Field Test (OFT), total distance in OFT (D) (one-way ANOVA followed by LSD’s multiple comparison tests, F _(2,24)_ = 0.490, n = 9, *p* = 0.619; Control vs LPS, *p* = 0.456; LPS vs LPS+MASM, *p* = 0.362); central distance in OFT (E) (one-way ANOVA followed by LSD’s multiple comparison tests, F _(2,24)_ = 0.203, n = 9, *p* = 0.818; Control vs LPS, *p* = 0.742; LPS VS LPS+MASM, *p* = 0.764). Data presented as mean ± SEM (*p < 0.05, **p < 0.01, ***p < 0.001). MASM, (6aS, 10S, 11aR, 11bR, 11cS)-10-methylamino-dodecahydro-3a, 7a-diazabenzo [de] anthracene-8-thione; LPS, lipopolysaccharide; ANOVA, analysis of variance; LSD, least significant difference; SEM, standard error of the mean.

LPS is a widely recognized inflammatory agent commonly used to induce depressive-like behaviors in experimental models. To investigate the potential effects of MASM on LPS-induced depressive-like behaviors in mice, we performed behavioral assessments, including the TST, FST, and OFT, as outlined in Fig. 1A. In both the TST (Fig. 1B) and FST (Fig. 1C), mice treated with LPS showed a marked increase in immobility time compared with the control group, confirming the successful induction of depressive-like behaviors. Notably, MASM treatment significantly reduced the immobility time in LPS-exposed mice, suggesting mitigation of despair-associated behaviors. Next, locomotor activity was evaluated using the OFT (Fig. 1D,E). Although no statistically significant differences were observed in overall locomotor activity among the control, LPS, and LPS+MASM groups, MASM appeared to enhance central excitability in the LPS-treated mice. These findings imply that MASM alleviates LPS-induced depressive-like behaviors.

### 3.2 Effects of MASM on the Expression of HO-1 Protein in the Hippocampus Induced by LPS

**Fig. 2.**
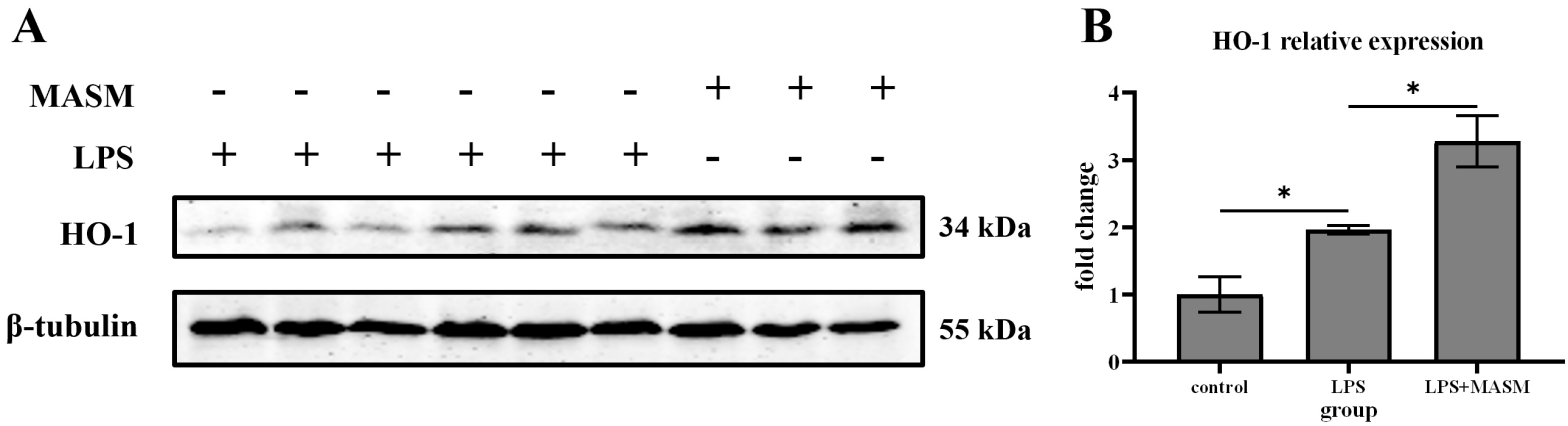
**MASM reduces LPS-induced effects on HO-1**. (A) Representative blots of HO-1 expression. (B) Representative bar graph of HO-1 expression analysis. Data presented as mean ± SEM (one-way ANOVA followed by LSD’s multiple comparison tests, F _(2,6)_ = 18.032, n = 3, *p* = 0.003; Control vs LPS, *p* = 0.045; LPS vs LPS+MASM, *p* = 0.014) (*p < 0.05). “+” means that the corresponding treatment is performed, while “-” means that there is no such treatment. HO-1, heme oxygenase-1.

HO-1, an inducible heat shock protein, is well-known for its potent anti-inflammatory and anti-apoptotic properties. To investigate whether MASM’s effects on depressive-like behaviors are associated with changes in HO-1 expression, we measured HO-1 levels in the hippocampus following LPS administration. Immunoblotting analysis revealed that HO-1 expression was significantly elevated in LPS-treated mice compared with the normal control group. Notably, MASM treatment further increased HO-1 expression in the LPS-treated mice (Fig. 2), suggesting that HO-1 may contribute to the protective effects of MASM against LPS-induced depressive-like behaviors.

### 3.3 MASM Suppresses LPS-Stimulated Increases in Proinflammatory Cytokine Levels in BV2 Cells

**Fig. 3.**
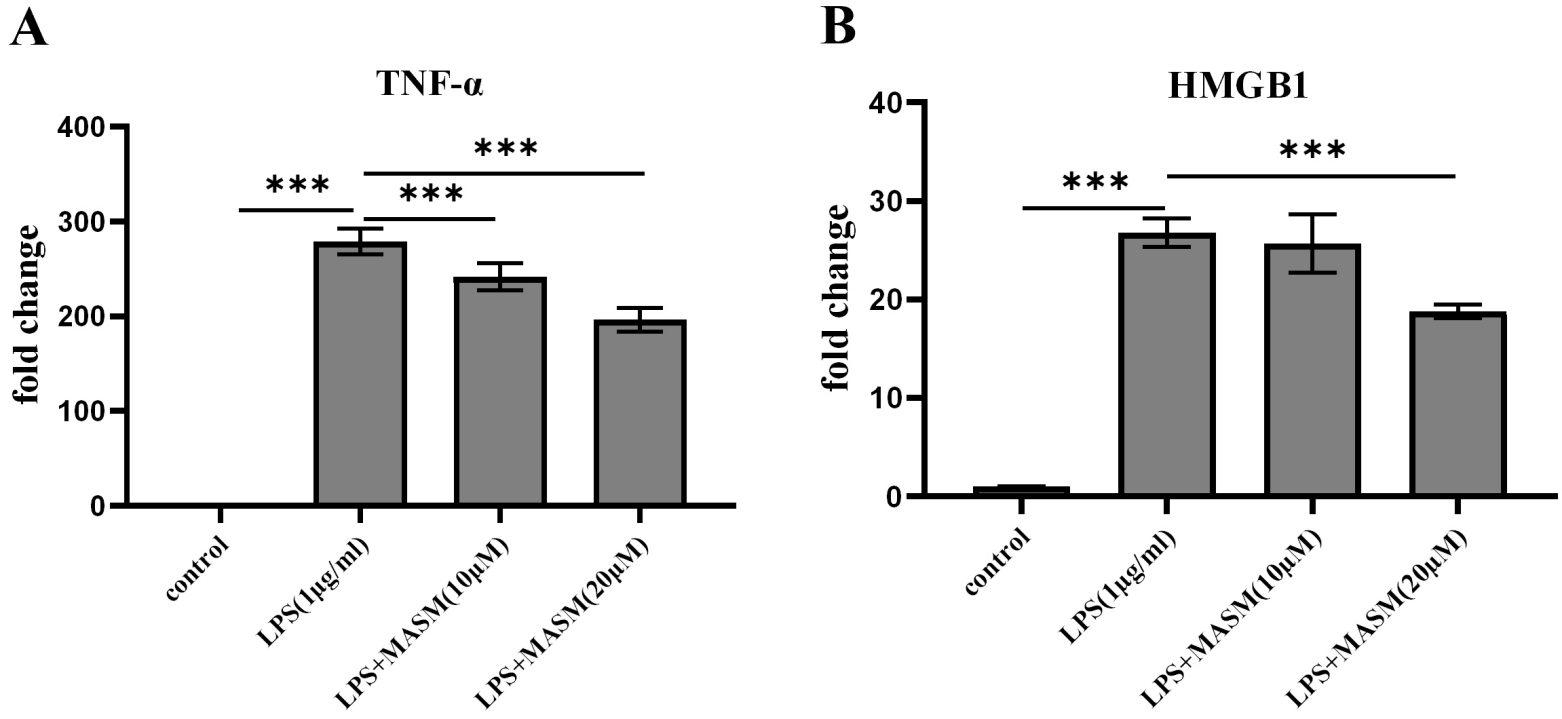
**MASM lowers LPS-induced proinflammatory cytokine levels in BV2 cells**. (A) Level of TNF-α (one-way ANOVA followed by LSD’s multiple comparison tests, F _(3,12)_ = 430.674, n = 4, *p* < 0.001; Control vs LPS, *p* < 0.001; LPS vs LPS+MASM (10 µM), *p* < 0.001; LPS+MASM (20 µM), *p* < 0.001). (B) Level of HMGB1 (one-way ANOVA followed by LSD’s multiple comparison tests, F _(3,8)_ = 50.011, n = 3, *p* < 0.001; Control vs LPS, *p* < 0.001; LPS vs LPS+MASM (10 µM), *p* = 0.654; LPS vs LPS+MASM (20 µM), *p* < 0.01). Data presented as mean ± SEM ( ***p < 0.001). HMGB1, high mobility group box 1.

To investigate whether MASM modulates LPS-induced proinflammatory responses, BV2 microglial cells were pretreated with either vehicle (1% DMSO, ST038-100ml, Beyotime) or MASM at concentrations of 10 µM and 20 µM for 24 hours, followed by stimulation with LPS (1 µg/mL) or PBS for an additional 24 hours. We then evaluated the effects of varying MASM concentrations on the levels of proinflammatory markers TNF-α and HMGB1. As shown in Fig. 3A,B, LPS treatment (1 µg/mL) significantly upregulated TNF-α and HMGB1 levels. In contrast, MASM at 10 µM markedly attenuated the LPS-induced increases in TNF-α and HMGB1 levels. Additionally, MASM at a higher concentration of 20 µM exhibited an even stronger anti-inflammatory effect, further reducing the levels of these proinflammatory markers.

### 3.4 MASM Regulated LPS-Stimulated Oxidative Stress in BV2 Cells

**Fig. 4.**
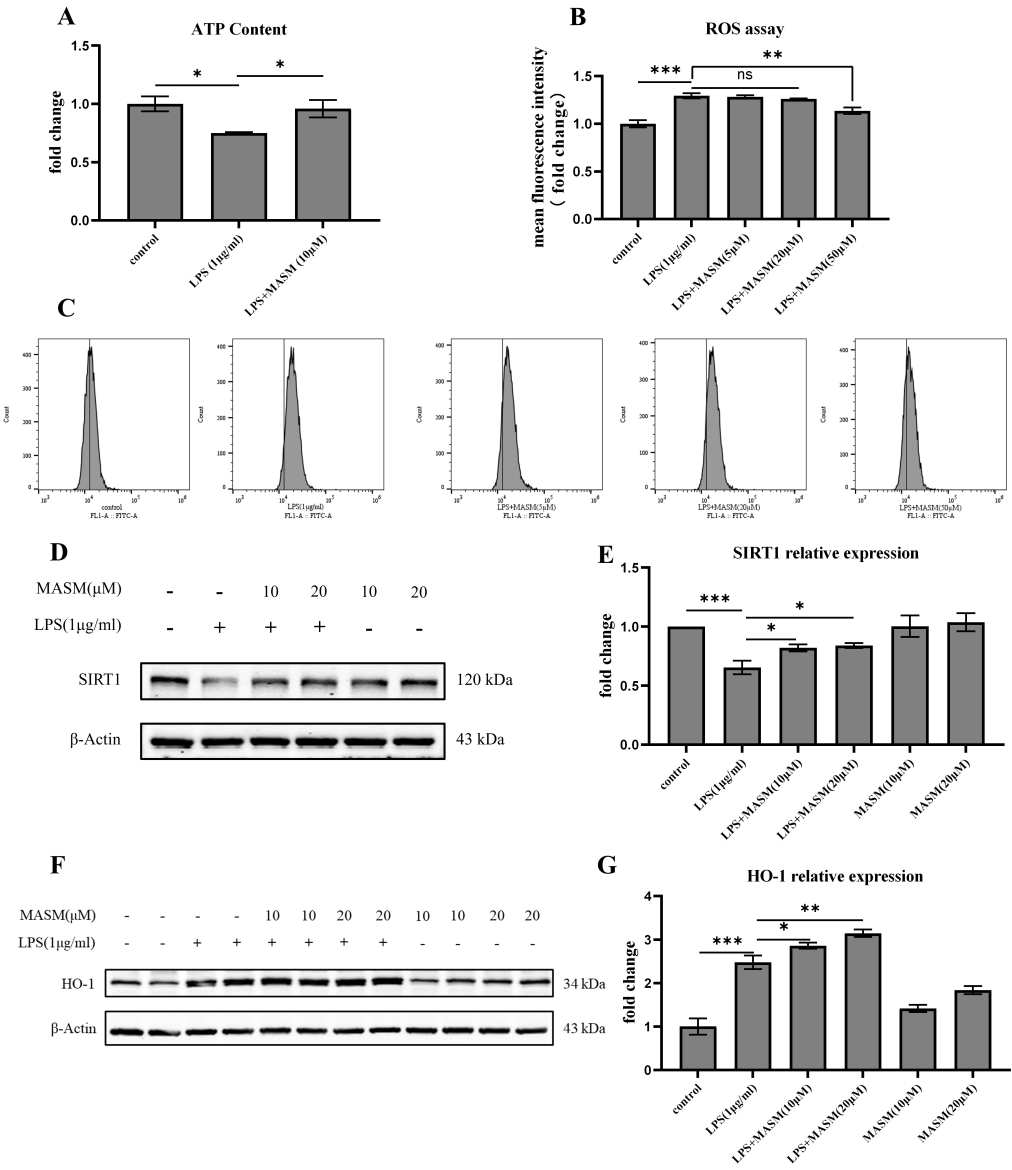
**MASM reduces LPS-Induced oxidative stress in BV2 cells**. (A) Relative concentration of intracellular ATP in BV2 cells treated with MASM (one-way ANOVA followed by LSD’s multiple comparison tests, F_(2,6)_ = 5.685, n = 3, *p* = 0.041; Control vs LPS, *p* = 0.020; LPS vs LPS+MASM (10 µM), *p* = 0.039). (B,C) Flow cytometry analysis of intracellular ROS production in BV2 cells treated with MASM (one-way ANOVA followed by LSD’s multiple comparison tests, F_(4,14)_ = 19.349, n = 3–4, *p* < 0.001; Control vs LPS, *p* < 0.001; LPS vs LPS+MASM (50 µM), *p* = 0.001). (D) Representative blots showing SIRT-1 expression. (E) Representative bar graph of SIRT-1 expression analysis (one-way ANOVA followed by LSD’s multiple comparison tests, F_(5,12)_ = 7.262, n = 3, *p* = 0.002; Control vs LPS, *p* < 0.001; LPS vs LPS+MASM (10 µM), *p* = 0.049; LPS vs LPS+MASM (20 µM), *p* = 0.035). (F) Representative blots showing HO-1 expression. (G) Representative bar graph of HO-1 expression analysis (one-way ANOVA followed by LSD’s multiple comparison tests, F_(5,18)_ = 48.175, n = 4, *p* < 0.001; Control vs LPS, *p* < 0.001; LPS vs LPS+MASM (10 µM), *p* = 0.039; LPS vs LPS+MASM (20 µM), *p* = 0.001). Data presented as mean ± SEM (*p < 0.05, **p < 0.01, ***p < 0.001). ROS, reactive oxygen species; ns, no significence; FL1-A, FL1-Area; FIT, fluorescein isothiocyanate.

To further investigate the potential antioxidative effects of MASM, we measured and analyzed levels of ATP, ROS, deacetylase SIRT-1, and HO-1 in BV2 cells. LPS (1 µg/mL) treatment led to a significant reduction in ATP levels and an increase in ROS production, indicating oxidative stress in the cells (Fig. 4A–C). MASM treatment at 10 µM effectively reversed the LPS-induced ATP reduction, while MASM at 50 µM significantly decreased ROS levels. Additionally, as shown in Fig. 4D–G, LPS (1 µg/mL) stimulation resulted in a notable decrease in SIRT-1 levels and an increase in HO-1 expression, both of which were ameliorated by MASM treatment at 10 or 20 µM. These findings suggest that MASM may alleviate oxidative stress in microglia induced by LPS.

### 3.5 MASM Reversed LPS-Induced Autophagy Inhibition in BV2 Cells

**Fig. 5.**
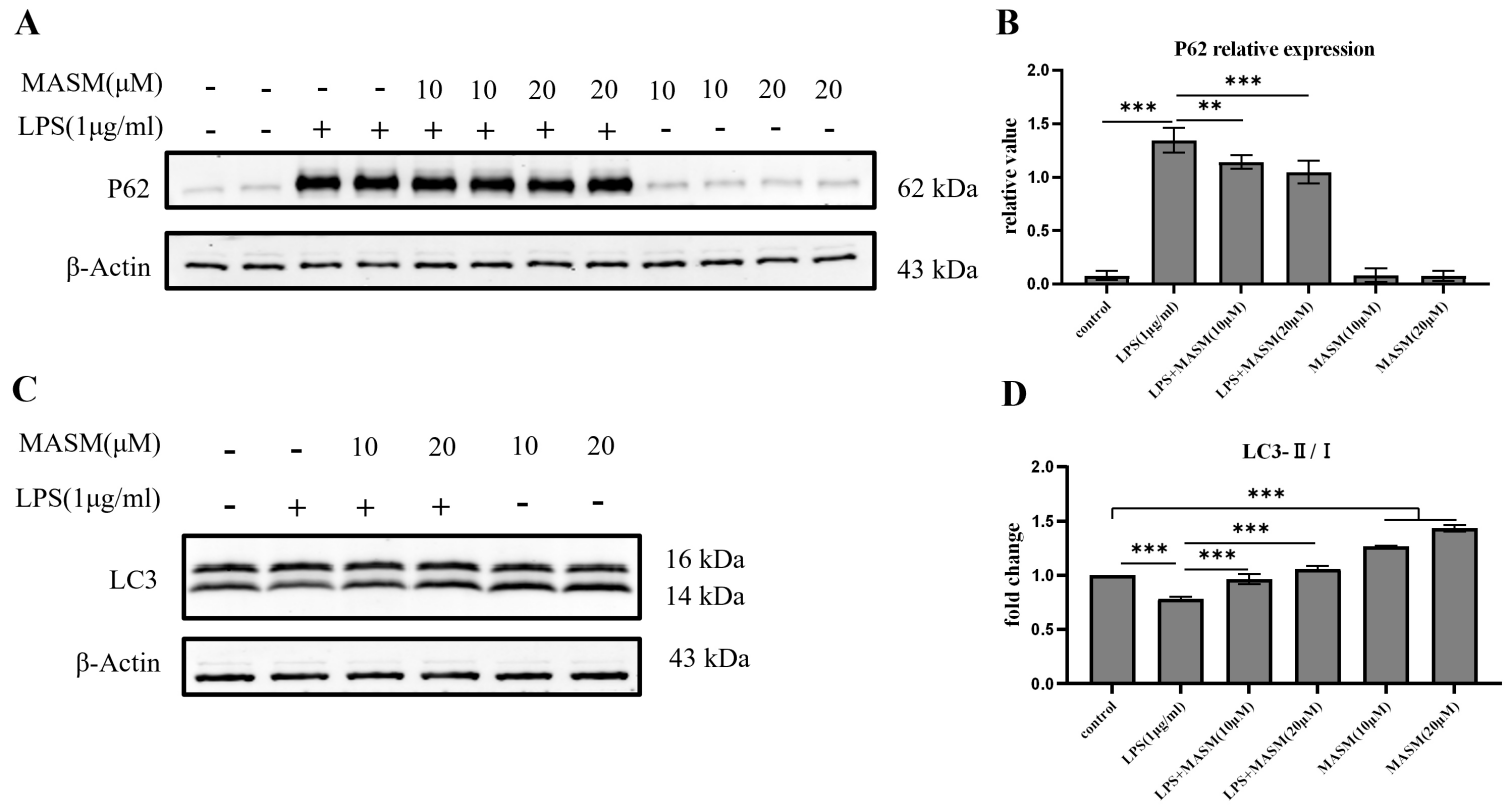
**MASM enhances autophagic flux in BV2 cells**. (A) Representative blots showing p62 expression. (B) Representative bar graph of p62 expression analysis (one-way ANOVA followed by LSD’s multiple comparison tests, F_(5,18)_ = 240.792, n = 4, *p* < 0.001; Control vs LPS, *p* < 0.001; LPS vs LPS+MASM (10 µM), *p* = 0.002; LPS vs LPS+MASM (20 µM), *p* < 0.001). (C) Representative blots showing LC3-Ⅱ/Ⅰ expression. (D) Representative bar graph of LC3-Ⅱ/Ⅰ expression analysis (one-way ANOVA followed by LSD’s multiple comparison tests, F_(5,12)_ = 81.805, n = 3, *p* < 0.001; Control vs LPS, *p* < 0.001; LPS vs LPS+MASM (10 µM), *p* < 0.001; LPS vs LPS+MASM (20 µM), *p* < 0.001. Data presented as mean ± SEM (**p < 0.01, *** *p* < 0.001). LC3, microtubule-associated protein 1 light chain 3.

Autophagy plays a crucial role in regulating microglial inflammation. However, it remains unclear whether MASM modulates LPS-induced microglial inflammation through autophagic mechanisms. To explore this, we analyzed the expression of the LC3 and p62, key markers of autophagic activity, using western blotting. Following stimulation with LPS (1 µg/mL), BV2 cells demonstrated a significant reduction in the LC3-II/I ratio and an increase in p62 expression, indicating disrupted autophagic activity. In contrast, pretreatment with MASM at 10 or 20 µM significantly elevated the LC3-II/I ratio and reduced p62 levels (Fig. 5A–D), suggesting a restoration of autophagic function. Under normal culture conditions, MASM treatment significantly increased LC3-II/I expression in BV2 cells, suggesting a potential role of MASM in promoting autophagic flux (Fig. 5D).

## 4. Discussion

The anti-inflammatory properties of MASM in peripheral inflammatory diseases have been extensively researched. Recent research underscores the critical role of neuroinflammation in the pathogenesis of depressive disorders. In this study, we investigated the effects of MASM on LPS-induced neuroinflammation and explored the underlying mechanisms involved. First, MASM significantly attenuated LPS-induced depressive-like behaviors by upregulating HO-1 expression. Second, MASM alleviated redox signaling changes, such as alterations in ATP, ROS, HO-1, and SIRT-1 levels, following LPS treatment. Additionally, MASM restored LPS-induced autophagy dysfunction. Collectively, these findings suggest that MASM can prevent neuroinflammation and alleviate depressive-like behaviors by modulating oxidative stress and autophagy.

Previous studies have demonstrated that intraperitoneal LPS administration effectively models neuroinflammation in animals. LPS treatment triggers a central inflammatory response characterized by elevated production of proinflammatory cytokines, microglial activation, and ROS generation, which can lead to depressive-like behaviors [24, 25]. In line with previous findings, our results demonstrate that LPS-induced neuroinflammation results in depressive-like behaviors, as evidenced by increased immobility in the TST and FST. Although no significant differences were observed in OFT between LPS-treated mice and the control group, indicating that mobility dysfunction or anxiety-like behavior in the model mice were not observed. Notably, MASM treatment significantly reduced LPS-induced depressive behaviors in these mice.

It is widely recognized that proinflammatory cytokines contribute to depression, as elevated cytokine levels have been observed in MDD patients [26]. Previous work from our group demonstrated that HMGB1 activates the kynurenine pathway in microglia, disrupting neurotransmitters and triggering depressive-like behaviors. Matrine, a compound with known anti-HMGB1 effects, also exhibits anti-inflammatory properties [27, 28]. In this study, we measured two representative cytokines, HMGB1 and TNF-α, in LPS-treated BV2 cells. MASM treatment significantly suppressed LPS-induced increases in TNF-α and HMGB1 levels, with a dose-dependent reduction in HMGB1, particularly at 20 µM. This aligns with previous studies showing that matrine suppresses TNF-α early in the inflammatory response, while HMGB1 suppression occurs later [29].

HO-1 is a well-known modulator of inflammation, involved in regulating the production of proinflammatory mediators and inhibiting the overproduction of M1 phenotype molecules [30]. In our study, compared with the control group, the LPS-treated group exhibited a significant increase in HO-1 expression, consistent with previous experimental findings [31]. This elevation in HO-1 levels is attributed to its protective role as an antioxidant and anti-inflammatory protein [32, 33]. Our *in vivo* experiments demonstrated that MASM promotes HO-1 expression, contributing to its antidepressant effects. Therefore, we established a neuroinflammation model in vitro to further investigate the antioxidant and anti-inflammatory effects of MASM.

Additionally, several studies have shown that MDD is accompanied by decreased antioxidant and increased ROS status [34]. Both preclinical and clinical evidence indicate that antidepressants can reduce oxidative stress by scavenging ROS and inhibiting oxidative pathways [35]. HO-1 is widely recognized as a key player in redox-regulated gene expression, responding to agents that generate ROS. In our study, MASM pretreatment significantly reduced oxidative stress in LPS-treated BV2 cells, as evidenced by decreased ROS and ATP levels. Moreover, SIRT-1—an nicotinamide adenine dinucleotide^+^ (NAD^+^)-dependent protein deacetylase involved in energy metabolism, stress response, inflammation, and redox homeostasis [36, 37]—was upregulated by MASM compared with LPS-treated cells.

Autophagy dysfunction is a major contributor to oxidative stress and chronic inflammation. LC3-II, a marker of autophagic activity, localizes to autophagic membranes, while p62, an autophagic substrate, monitors autophagic turnover [38]. Our results showed that MASM increased LC3-II expression and decreased p62 levels, indicating a restoration of LPS-induced autophagic flux in BV2 microglial cells. Similarly, MASM reportedly exerts anticancer effects by promoting autophagy and inhibiting oxidative stress [39]. Our findings reveal that MASM plays a critical role in regulating redox homeostasis and autophagy in the context of depression.

Despite these promising findings, there are limitations to our research. Future studies should explore the protective effects of MASM through inhibition of HO-1, using HO-1 inhibitors or gene silencing approaches. Additionally, the role of transcription factor Nrf-2 in MASM-mediated HO-1 upregulation should be investigated to better understand the underlying mechanisms. The present study provides an initial exploration of the autophagy-enhancing effects of MASA, concentrating exclusively on changes in cytoplasmic autophagic flux. The subcellular localization of autophagic processes has not yet been examined in detail. In future studies, we intend to focus on selective autophagy pathways, such as mitophagy, guided by findings from transmission electron microscopy (TEM). Although MASM demonstrates strong anti-inflammatory effects and offers the advantages typical of natural products, such as enhanced bioavailability and low toxicity, it shows significant potential for clinical translation, particularly for patients with high-inflammatory depression. However, we did not explore the optimal effective dosage *in vivo* or investigate further molecular mechanisms. Therefore, future studies are required to validate MASM’s clinical applicability through additional experiments, including dosage optimization and mechanistic studies, such as examining the causal relationship between oxidative stress and autophagy.

## 5. Conclusion

In summary, this study revealed for the first time that MASM significantly alleviates acute depressive-like behaviors, decreases oxidative stress, and promotes autophagy in both an LPS-induced acute depression model and LPS-treated BV2 cells. Furthermore, MASM inhibits LPS-induced inflammatory responses by regulating the expression of HO-1 and SIRT-1. Due to its demonstrated effectiveness and safety, MASM shows potential as a novel therapeutic approach for treating inflammation-related neurological disorders, including MDD.

## Availability of Data and Materials

The datasets used or analyzed during the current study are available from the corresponding author on reasonable request.

## References

[1] Marwaha S, Palmer E, Suppes T, Cons E, Young AH, Upthegrove R (2023). Novel and emerging treatments for major depression. *Lancet (London, England)*.

[2] Monroe SM, Harkness KL (2022). Major Depression and Its Recurrences: Life Course Matters. *Annual Review of Clinical Psychology*.

[3] Liu S, Xu S, Wang Z, Guo Y, Pan W, Shen Z (2018). Anti-Depressant-Like Effect of Sinomenine on Chronic Unpredictable Mild Stress-Induced Depression in a Mouse Model. *Medical Science Monitor: International Medical Journal of Experimental and Clinical Research*.

[4] Beurel E, Toups M, Nemeroff CB (2020). The Bidirectional Relationship of Depression and Inflammation: Double Trouble. *Neuron*.

[5] Colasanto M, Madigan S, Korczak DJ (2020). Depression and inflammation among children and adolescents: A meta-analysis. *Journal of Affective Disorders*.

[6] Zhang Y, Wang J, Ye Y, Zou Y, Chen W, Wang Z (2023). Peripheral cytokine levels across psychiatric disorders: A systematic review and network meta-analysis. *Progress in Neuro-psychopharmacology & Biological Psychiatry*.

[7] Ng A, Tam WW, Zhang MW, Ho CS, Husain SF, McIntyre RS (2018). IL-1β, IL-6, TNF- α and CRP in Elderly Patients with Depression or Alzheimer’s disease: Systematic Review and Meta-Analysis. *Scientific Reports*.

[8] Han S, Li XX, Wei S, Zhao D, Ding J, Xu Y (2023). Orbitofrontal cortex-hippocampus potentiation mediates relief for depression: A randomized double-blind trial and TMS-EEG study. *Cell Reports. Medicine*.

[9] Price RB, Duman R (2020). Neuroplasticity in cognitive and psychological mechanisms of depression: an integrative model. *Molecular Psychiatry*.

[10] Lukens JR, Eyo UB (2022). Microglia and Neurodevelopmental Disorders. *Annual Review of Neuroscience*.

[11] Wang H, He Y, Sun Z, Ren S, Liu M, Wang G (2022). Microglia in depression: an overview of microglia in the pathogenesis and treatment of depression. *Journal of Neuroinflammation*.

[12] Fang S, Wu Z, Guo Y, Zhu W, Wan C, Yuan N (2023). Roles of microglia in adult hippocampal neurogenesis in depression and their therapeutics. *Frontiers in Immunology*.

[13] Brown SJ, Huang XF, Newell KA (2021). The kynurenine pathway in major depression: What we know and where to next. *Neuroscience and Biobehavioral Reviews*.

[14] Huang J, Xu H (2016). Matrine: Bioactivities and Structural Modifications. *Current Topics in Medicinal Chemistry*.

[15] Sun D, Wang J, Yang N, Ma H (2016). Matrine suppresses airway inflammation by downregulating SOCS3 expression via inhibition of NF-κB signaling in airway epithelial cells and asthmatic mice. *Biochemical and Biophysical Research Communications*.

[16] Chu Y, Jing Y, Zhao X, Wang M, Zhang M, Ma R (2021). Modulation of the HMGB1/TLR4/NF-κB signaling pathway in the CNS by matrine in experimental autoimmune encephalomyelitis. *Journal of Neuroimmunology*.

[17] Yang Y, Xiu J, Zhang X, Zhang L, Yan K, Qin C (2012). Antiviral effect of matrine against human enterovirus 71. *Molecules (Basel, Switzerland)*.

[18] Hu H, Wang S, Zhang C, Wang L, Ding L, Zhang J (2010). Synthesis and in vitro inhibitory activity of matrine derivatives towards pro-inflammatory cytokines. *Bioorganic & Medicinal Chemistry Letters*.

[19] Fan ZY, Chen YP, Chen L, Zhang XQ, Chen LL, Lu B (2022). The matrine derivate MASM inhibits astrocyte reactivity and alleviates experimental autoimmune encephalomyelitis in mice. *International Immunopharmacology*.

[20] Xu J, Qi Y, Xu WH, Liu Y, Qiu L, Wang KQ (2016). Matrine derivate MASM suppresses LPS-induced phenotypic and functional maturation of murine bone marrow-derived dendritic cells. *International Immunopharmacology*.

[21] Zhang Y, Liu L, Peng YL, Liu YZ, Wu TY, Shen XL (2014). Involvement of inflammasome activation in lipopolysaccharide-induced mice depressive-like behaviors. *CNS Neuroscience & Therapeutics*.

[22] Armario A (2021). The forced swim test: Historical, conceptual and methodological considerations and its relationship with individual behavioral traits. *Neuroscience and Biobehavioral Reviews*.

[23] Wei JP, Wen W, Dai Y, Qin LX, Wen YQ, Duan DD (2021). Drinking water temperature affects cognitive function and progression of Alzheimer’s disease in a mouse model. *Acta Pharmacologica Sinica*.

[24] O’Connor JC, Lawson MA, André C, Moreau M, Lestage J, Castanon N (2009). Lipopolysaccharide-induced depressive-like behavior is mediated by indoleamine 2,3-dioxygenase activation in mice. *Molecular Psychiatry*.

[25] Zhang JC, Wu J, Fujita Y, Yao W, Ren Q, Yang C (2014). Antidepressant effects of TrkB ligands on depression-like behavior and dendritic changes in mice after inflammation. *The International Journal of Neuropsychopharmacology*.

[26] Köhler CA, Freitas TH, Maes M, de Andrade NQ, Liu CS, Fernandes BS (2017). Peripheral cytokine and chemokine alterations in depression: a meta-analysis of 82 studies. *Acta Psychiatrica Scandinavica*.

[27] Huang X, Wang B, Yang J, Lian YJ, Yu HZ, Wang YX (2023). HMGB1 in depression: An overview of microglial HMBG1 in the pathogenesis of depression. *Brain, Behavior, & Immunity - Health*.

[28] Wang B, Huang X, Pan X, Zhang T, Hou C, Su WJ (2020). Minocycline prevents the depressive-like behavior through inhibiting the release of HMGB1 from microglia and neurons. *Brain, Behavior, and Immunity*.

[29] Zhang B, Liu ZY, Li YY, Luo Y, Liu ML, Dong HY (2011). Antiinflammatory effects of matrine in LPS-induced acute lung injury in mice. *European Journal of Pharmaceutical Sciences: Official Journal of the European Federation for Pharmaceutical Sciences*.

[30] Choubey P, Kwatra M, Pandey SN, Kumar D, Dwivedi DK, Rajput P (2019). Ameliorative effect of fisetin against lipopolysaccharide and restraint stress-induced behavioral deficits via modulation of NF-κB and IDO-1. *Psychopharmacology*.

[31] Huang X, Fei GQ, Liu WJ, Ding J, Wang Y, Wang H (2020). Adipose-derived mesenchymal stem cells protect against CMS-induced depression-like behaviors in mice via regulating the Nrf2/HO-1 and TLR4/NF-κB signaling pathways. *Acta Pharmacologica Sinica*.

[32] Wang J, Behl T, Rana T, Sehgal A, Wal P, Saxena B (2024). Exploring the pathophysiological influence of heme oxygenase-1 on neuroinflammation and depression: A study of phytotherapeutic-based modulation. *Phytomedicine: International Journal of Phytotherapy and Phytopharmacology*.

[33] Dang R, Wang M, Li X, Wang H, Liu L, Wu Q (2022). Edaravone ameliorates depressive and anxiety-like behaviors via Sirt1/Nrf2/HO-1/Gpx4 pathway. *Journal of Neuroinflammation*.

[34] Bhatt S, Nagappa AN, Patil CR (2020). Role of oxidative stress in depression. *Drug Discovery Today*.

[35] Xu Y, Wang C, Klabnik JJ, O’Donnell JM (2014). Novel therapeutic targets in depression and anxiety: antioxidants as a candidate treatment. *Current Neuropharmacology*.

[36] Nandave M, Acharjee R, Bhaduri K, Upadhyay J, Rupanagunta GP, Ansari MN (2023). A pharmacological review on SIRT 1 and SIRT 2 proteins, activators, and inhibitors: Call for further research. *International Journal of Biological Macromolecules*.

[37] Beegum F, P V A, George KT, K P D, Begum F, Krishnadas N (2022). Sirtuins as therapeutic targets for improving delayed wound healing in diabetes. *Journal of Drug Targeting*.

[38] Loos B, du Toit A, Hofmeyr JHS (2014). Defining and measuring autophagosome flux-concept and reality. *Autophagy*.

[39] Zou Y, Sarem M, Xiang S, Hu H, Xu W, Shastri VP (2019). Autophagy inhibition enhances Matrine derivative MASM induced apoptosis in cancer cells via a mechanism involving reactive oxygen species-mediated PI3K/Akt/mTOR and Erk/p38 signaling. *BMC Cancer*.

